# The molecular mechanism of circRNA_0021727 in inflammation of eosinophilic chronic rhinosinusitis

**DOI:** 10.3389/fimmu.2026.1737875

**Published:** 2026-03-27

**Authors:** MuHan Shi, Tong Guo, Nan Lin, XiuQuan Liu, XinZhu Wang, DeYun Wang, LiSheng Yu, Min Wang

**Affiliations:** 1 Department of Otorhinolaryngology Head and Neck Surgery, Peking University People’s Hospital, Beijing, China; 2 Department of Otolaryngology, Yong Loo Lin School of Medicine, National University of Singapore, Singapore, Singapore

**Keywords:** ADAM12, circRNA, eosinophilic chronic rhinosinusitis, microRNA, nasal polyp

## Abstract

**Purpose:**

Eosinophilic chronic rhinosinusitis (ECRS) is characterized by severe symptoms and a high recurrence rate, yet its pathogenesis remains unclear and difficult to manage with current treatments. This study aims to investigate the role of circRNA_0021727 in ECRS-related inflammation, as well as the regulatory interactions among circRNA_0021727, miRNA_145_5p, and ADAM12 in ECRS.

**Methods:**

Primary nasal epithelial cells (PNECs) were isolated from nasal polyp tissues of 10 ECRS patients and inferior turbinate tissues of 10 nasal septum deviation patients for differential expression analysis. Primary cell cultures were established for overexpression and knockdown of circRNA_0021727 and miRNA_145_5p, which were generated to assess ADAM12 expression. An inflammatory model was induced using IL-13 treatment, and the expression levels of GM-CSF, EOTAXIN, and MUC5AC were measured.

**Results:**

In ECRS epithelium, circRNA_0021727 and ADAM12 were upregulated, whereas miRNA_145_5p was downregulated. Overexpression of circRNA_0021727 led to decreased miRNA-145-5p and increased ADAM12 expression, whereas knockdown of circRNA_0021727 had the opposite effect. Similarly, overexpression of miRNA_145_5p resulted in decreased ADAM12 levels, while its knockdown increased ADAM12 expression. After IL-13 treatment, GM-CSF, EOTAXIN, and MUC5AC expression levels were elevated in circRNA_0021727-overexpressing cells and reduced in knockdown cells. Conversely, miRNA_145_5p overexpression decreased these inflammatory markers, while knockdown increased their levels.

**Conclusion:**

The circRNA_0021727-miRNA-145-5p-ADAM12 regulatory axis plays a role in ECRS pathogenesis. circRNA_0021727 promotes ECRS-related inflammation, suggesting its potential as a therapeutic target for managing the disease.

## Introduction

Chronic rhinosinusitis (CRS) is a chronic inflammatory disease of the nasal cavity and paranasal sinus mucosa (1). The Global Allergy and Asthma European Network (GA2LEN), according to the criteria of the European Position Paper on Rhinosinusitis and Nasal Polyps (EPOS), found in a survey of 12 European countries that the overall prevalence of CRS was 10.9% (2), indicating a large patient population. Among CRS cases, the treatment of eosinophilic chronic rhinosinusitis (ECRS) is particularly challenging. Patients with ECRS often have comorbid conditions such as bronchial asthma, allergic rhinitis, and aspirin intolerance, further complicating management. Although surgical treatment is often successful and medical therapy is standardized, many patients will still experience recurrence after surgery (3). Therefore, ECRS remains a major challenge in treatment. A significant increase in peripheral blood and tissue eosinophil levels is a hallmark of ECRS. Various cytokines play essential roles in its pathogenesis. IL-3, IL-5, and Granulocyte-macrophage Colony Stimulating Factor (GM-CSF) are crucial for eosinophil development and release from the bone marrow into the peripheral blood, contributing to ECRS progression (4). EOTAXIN recruits and activates eosinophils, driving their migration to the inflamed sites and amplifying the inflammatory response (5). IL-13, a key cytokine in allergic respiratory diseases, has been implicated in both mouse models and human studies (6, 7). Along with IL-4, it activates multiple cell types, disrupts barrier function, and promotes inflammation (8). In ECRS cell culture, IL-13 increases β-catenin expression, further driving disease progression (9). Additionally, IL-13 upregulates MUC5AC in goblet cells, exacerbating nasal mucosal inflammation (10).

CircRNA, an endogenous non-coding RNA with a closed circular structure, lacks a 5’-end cap and a 3'-end polyadenylation, making it resistant to RNases, highly stable, and evolutionarily conserved (11). These properties make circRNA a promising diagnostic marker or therapeutic target. Functionally, circRNA can act as a competitive miRNA sponge, reducing available miRNA molecules, inhibiting target mRNA degradation, and promoting gene upregulation (12, 13). In our previous study, we performed whole-transcriptome sequencing on nasal polyps from patients with chronic rhinosinusitis with nasal polyps (CRSwNP) and compared them to inferior turbinate tissues from patients with nasal septum deviation, identifying differentially expressed RNAs. The results revealed that circRNA_0021727 and ADAM12 were highly expressed in CRSwNP, whereas miRNA_145_5p was downregulated (14). Additionally, circRNA_0021727 expression positively correlated with eosinophil parameters, TNF-α, IFN-γ, and tissue eosinophil count. Sequence analysis suggested a potential regulatory axis involving circRNA_0021727, miRNA_145_5p, and ADAM12. In this study, we collected clinical tissue specimens from patients with ECRS and nasal septum deviation, analyzed expression differences, validated the regulatory pathway in primary nasal epithelial cells, and investigate the effect of circRNA_0021727 on inflammation in ECRS. These findings provide a foundation for identifying novel diagnostic markers and therapeutic targets.

## Materials and methods

### Patients and sample collection

The nasal polyp tissues of 10 ECRS patients who met the EPOS2020 criteria, that is, the average number of eosinophils in each of the 5 high-power microscopic fields of the polyp tissue was > 10 (1), were collected and used as the experimental group. The control group consisted of the inferior turbinate tissues collected from 10 patients with nasal septum deviation, who were excluded from allergic rhinitis through allergen test. These 10 patients all underwent nasal septum deviation correction surgery under nasal endoscopy, and CT scans confirmed that there were no inflammatory manifestations in both the nasal cavity and paranasal sinuses. All the subjects read and understood the patient instructions and signed the informed consent forms.

### Cell culture and treatment

All tissue samples were respectively subjected to primary culture. Briefly, tissues were repeatedly rinsed with sterile PBS containing 1% penicillin-streptomycin (P/S) solution (Thermo Fisher Scientific, USA). Tissues were then minced into small pieces (approx. 1–2 mm³) and incubated overnight at 4 °C in a dissociation medium containing 0.1% proteinase K (Sigma-Aldrich, USA) in BEGM medium. Following digestion, cells were dislodged, centrifuged at 300xg for 5 minutes at 4 °C, and the cell pellet was resuspended. Cells were then seeded onto collagen-coated culture dishes with BEGM medium (Lonza, USA) supplemented with 1% P/S. The medium was changed every two days until cells reached 70-80% confluence, as assessed by visual inspection under a phase-contrast microscope, before subculture. Cell viability was routinely monitored by trypan blue exclusion. For certain experiments, IL-13 (R&D Systems, USA) was added to the medium at a final concentration of 20 ng/mL. Cells were cultured in an incubator at 37 °C with 5% CO2. For the 14-day stimulation protocol, IL-13-containing medium was replenished every two days.

### Transfection

The circRNA lentiviral vector and its negative control viral vector were purchased from Genechem (Shanghai, China). The primary cells were plated at an appropriate density. The lentiviral vector contains the puromycin resistance gene and the fluorescent protein gene. After adding the virus (MOI = 10) and transfection reagent (Polybrene, 5 µg/mL) and incubating for 48 hours, puromycin treatment (2 µg/mL) is performed once microscopic examination confirms that more than 80% of the cells express the fluorescent protein. Following two cell passages, successfully transfected cells with normal proliferation can be obtained. The miRNA mimics, inhibitors (final concentration 50 nM), and negative control substances were purchased from Thermo Fisher Scientific (Waltham, MA, USA). According to the manufacturer’s operation instructions, the miRNA mimics and inhibitors were transfected into the cells using Lipofectamine 3000 (Thermo Fisher Scientific, USA) at a final concentration of 5 µL/mL.

### Real−time quantitative PCR

Total RNA of the primary cells was extracted using Trizol reagent (RNAiso Plus, TAKARA) following the manufacturer’s protocol. The RNA extracts were reverse transcribed into cDNA and then quantified by SYBR Green assay (Toyobo, Japan). GAPDH and U6 were used as endogenous references for mRNAs and miRNAs, respectively. Primer of miRNA_145_5p was purchased from Thermo Fisher Scientific (Waltham, MA, USA). Other primer sequences are listed in **Table 1**. All primer sequences are presented in. Each assay was run in triplicate, and relative expression was calculated using the comparative cycle threshold method.

**Table 1:** Sequences of the primers used for qRT-PCR.

Primer(5’-3’)	Sequence
GMCSF-F	ATGTGGCTGCAGAGCCTGCTGC
GMCSF-R	CTCCCAGCAGTCAAAGGG
EOTAXIN-F	GTCACAGTTGCTGGGAGTCAT
EOTAXIN-R	AAGGGTTGCTACGGAGAGGA
MUC5AC-F	CAGCCACGTCCCCTTCAATA
MUC5AC-R	ACCGCATTTGGGCATCC
GAPDH-F	CCTCTGACTTCAACAGCGACAC
GAPDH-R	TGGTCCAGGGGTCTTACTCC
circRNA_0021727-F	TGGTCCAGGGGTCTTACTCC
circRNA_0021727-R	GAGGAAGAAGAGACCCCACA
ADAM12-F	CGCTCGAAATTACACGGGTC
ADAM12-R	ACACGTGCTGAGACTGACTG

### Enzyme−linked immunosorbent assay

The levels of GM-CSF and EOTAXIN in the cell supernatant were precisely measured using high-quality commercial ELISA kits sourced from R&D Systems (Minneapolis, MN, USA). For the detection of MUC5AC, a highly specific Human Mucin-5 subtype AC ELISA kit was provided by Blue Gene Biotech (Shanghai, China). All the detections were carried out in strict accordance with the detailed instructions provided by the respective manufacturers to ensure the accuracy and reliability of the results.

### Western blot

Total proteins were extracted from the primary cells with the application of RIPA lysis buffer (Biodee, China). The protein concentration was determined by utilizing the BCA Protein Assay Kit (Thermo Fisher Scientific, USA). A total amount of 20 µg of the extracted proteins was loaded onto a sodium dodecylsulfate–polyacrylamide gel electrophoresis (SDS-PAGE) gel. Subsequently, the proteins were transferred onto a polyvinylidene fluoride (PVDF) membrane. To block the membrane, 5% skimmed milk was employed, and the membrane was incubated at room temperature for one hour. Following the blocking step, the membranes were then incubated with the primary antibodies overnight. On the next day, after being washed three times with a tris-buffered saline buffer that contained 0.1% Tween 20, the membranes were incubated with the corresponding horse radish peroxidase-conjugated secondary antibodies at room temperature for another hour. The primary antibodies utilized in this present study were anti-ADAM12, purchased from Abcam (Cambridge, UK), and anti-GAPDH (from Cell Signaling Technology). The density of the protein bands was quantified by using the GS-900TM Calibrated Densitometer (Bio-Rad) and further analyzed through the use of Image Lab software (Bio Rad).

### Statistical analysis

All data were presented as mean ± SD and analyzed by SPSS 26.0 software (IBM Corp.) and GraphPad Prism 6.0 software (GraphPad Software, Inc.). Data were analyzed by 2-way analysis of variance or Student t test analysis. The statistical difference was considered as significant when P value is < 0.05.

## Result

### Detection of differential RNA expressions

**Figure 1:**
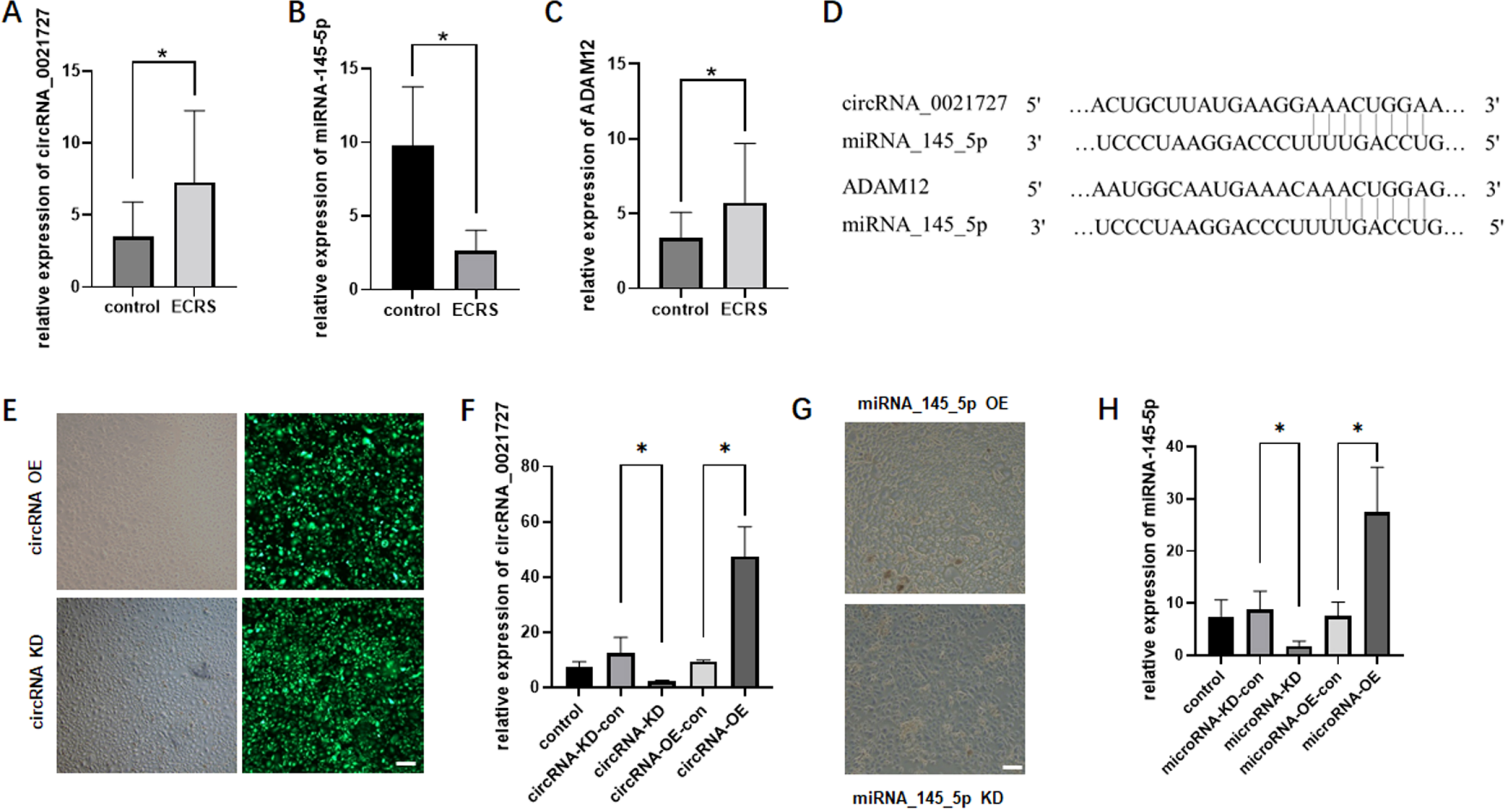
The relative expression levels of circRNA_0021727, miRNA_145_5p, and ADAM12 in patients with ECRSwNP, and construction of overexpression (OE) and knockdown (KD) cells for circRNA_0021727 and miRNA_145_5p. **(A–C)**. The comparison of the expression differences of circRNA_0021727, miRNA_145_5p, and ADAM12 between ECRSwNP and non-sinusitis samples. **(A)** circRNA_0021727 is significantly upregulated in ECRS. **(B)** miRNA_145_5p is significantly downregulated in ECRS. **(C)** ADAM12 is significantly upregulated in ECRS. **(D)** Bioinformatic prediction of miR-145-5p binding sites in circRNA_0021727 and ADAM12 3′UTR. **(E)** The relative expression levels of circRNA_0021727 in the constructed circRNA_0021727 OE and KD cells. The control group consists of untreated primary cells. **(F)** The results of circRNA_0021727 overexpression (OE) and knockdown (KD) cells after transfection, vector contains the fluorescent protein gene, with a scale bar of 50 μm. The lentiviral vector contains the fluorescent protein gene, cells expressing the fluorescent protein indicate successful transfection. **(G)** The results of miRNA_145_5p overexpression (OE) and knockdown (KD) cells after transfection, with a scale bar of 50 μm. **(H)** The relative expression levels of miRNA_145_5p in the constructed miRNA_145_5p OE and KD cells. The control group consists of untreated primary cells.

Through RT-qPCR detection, it was found circRNA_0021727 was significantly up-regulated in the nasal polyp tissues of ECRS patients as compared to controls, but miRNA_145_5p was significantly down-regulated and ADAM12 was significantly up-regulated (P<0.05), indicating that the differences were statistically significant (**Figures 1A–C**).We predicted a specific and functional binding site for miRNA_145_5p in the 3’ UTR of ADAM12 mRNA. Additionally, based on the circRNA_0021727 sequence acquired from circBase, we identified putative binding sites for miRNA_145_5p within circRNA_0021727 (**Figure 1D**).

### Construction of overexpression and knockdown cells for circRNA_0021727 and miRNA_145_5p

Primary epithelial cells were transfected with lentiviruses carrying genes for overexpressing or knocking down circRNA_0021727, followed by puromycin selection. The transfection efficiency of the cells could be observed under a fluorescence microscope (**Figure 1E**). When the cell confluence reached 80%, the cells were collected, and RNA was extracted. After reverse transcription, the expression level of circRNA_0021727 was detected by RT-qPCR. The results showed that the expression level of circRNA_0021727 in the OE group was significantly higher than that in the control group, while the expression level in the KD group was significantly lower than that in the control group, and the differences were statistically significant (**Figure 1F**).

Primary cells were transfected with miRNA_145_5p mimic and inhibitor to construct OE and KD models of miRNA_145_5p respectively (**Figure 1G**). When the cell confluence reached 80%, the cells were collected, and RNA was extracted. After reverse transcription, the expression level of miRNA_145_5p was detected by RT-qPCR. The results showed that the expression level of miRNA_145_5p in the OE group was significantly higher than that in the control group, while the expression level in the KD group was significantly lower than that in the control group, and the differences were statistically significant (**Figure 1H**).

### Change of inflammatory factors

**Figure 2:**
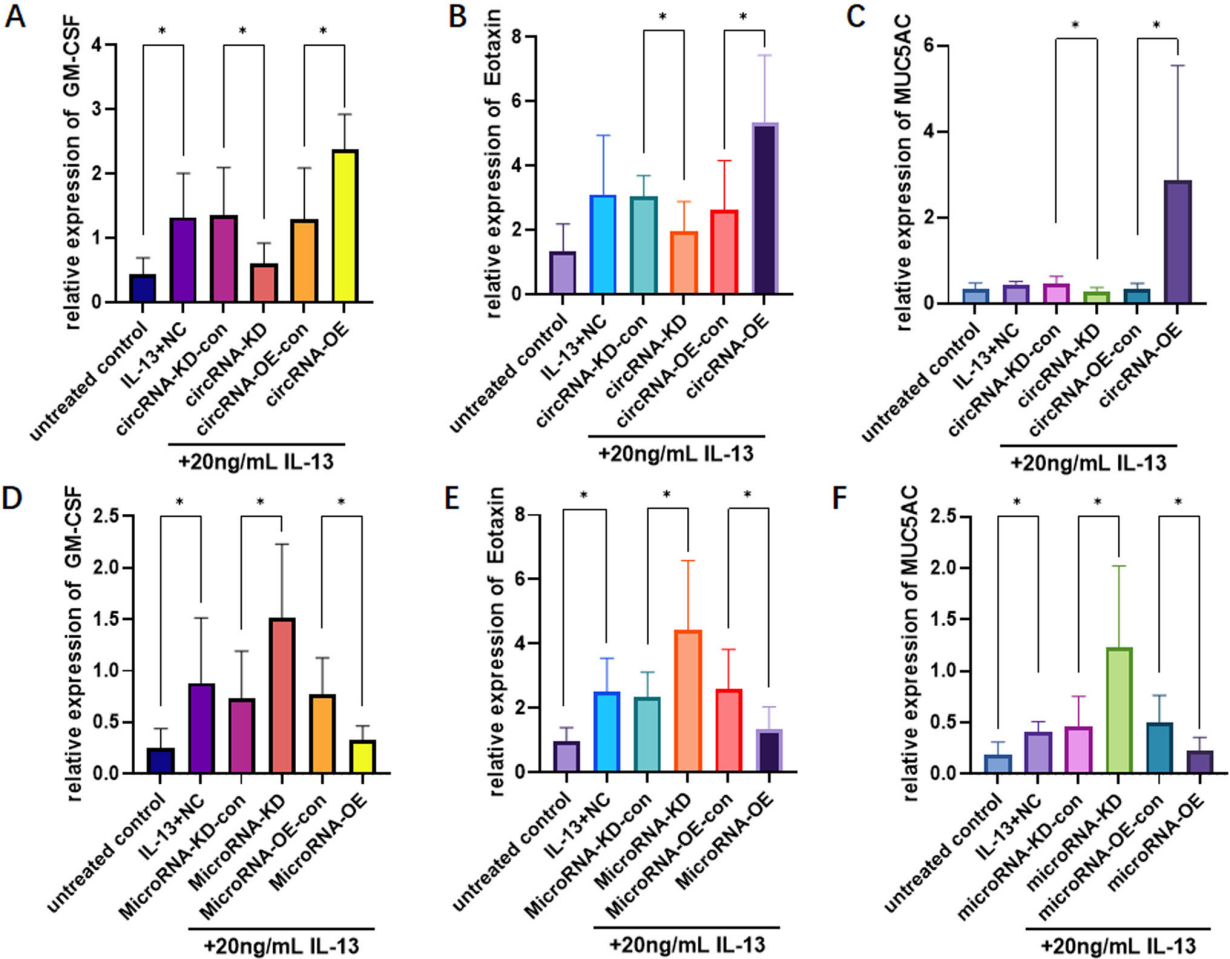
Relative mRNA expression levels of GM-CSF, EOTAXIN, and MUC5AC in circRNA_0021727 and miRNA_145_5p overexpression (OE) and knockdown (KD) cells under IL-13 conditions. In circRNA_0021727 OE cells, GM-CSF, EOTAXIN, and MUC5AC levels were significantly higher than in controls, while in circRNA_0021727 KD cells, these levels were significantly lower **(A–C)**. Conversely, in miRNA_145_5p OE cells, GM-CSF, EOTAXIN, and MUC5AC levels were significantly lower than in controls, while in miRNA_145_5p KD cells, these levels were significantly higher **(D–F)**. OE, overexpression; KD, knockdown; NC, negative control. *P<0.05.

After 14 days of treatment with IL-13, the OE and KD cells of circRNA_0021727 and miRNA_145_5p were collected. RNA and proteins were extracted from these cells. The extracted RNA was reverse-transcribed, and then the expression levels of mRNAs corresponding to GM-CSF, EOTAXIN, and MUC5AC in each group were detected by RT-qPCR. The results indicated that the expression levels of GM-CSF, EOTAXIN, and MUC5AC in the circRNA_0021727 OE cells were significantly higher than those in the control group, while the expression levels in the circRNA_0021727 KD cells were significantly lower than those in the control group (**Figures 2A–C**). In the miRNA_145_5p OE cells, the expression levels of GM-CSF, EOTAXIN, and MUC5AC were significantly lower than those in the control group, whereas in the miRNA_145_5p KD cells, the expression levels were significantly higher than those in the control group (**Figures 2D–F**).

**Figure 3:**
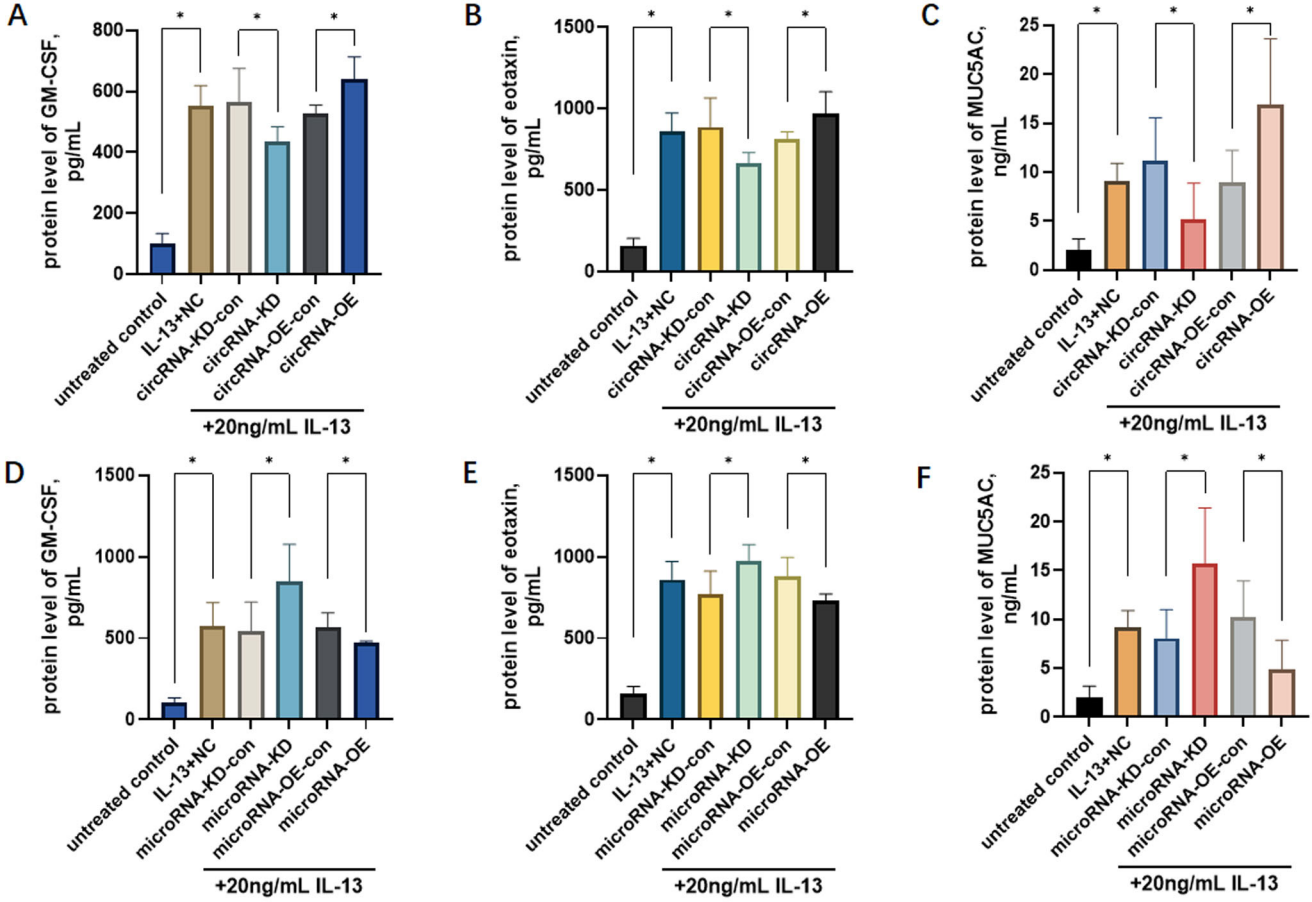
The protein levels of GM-CSF, EOTAXIN, and MUC5AC in the overexpression and knockdown cells of circRNA_0021727 and miRNA_145_5p under IL-13 condition. In circRNA_0021727 OE cells, GM-CSF, EOTAXIN, and MUC5AC levels were significantly higher than in controls, and these levels in circRNA_0021727 KD cells were significantly lower **(A–C)**. Conversely, in miRNA_145_5p OE cells, these levels were significantly lower than in controls, while in miRNA_145_5p KD cells, they were significantly higher **(D–F)**. OE, overexpression, KD, knockdown; NC, negative control. *P<0.05.

The protein contents of GM-CSF, EOTAXIN, and MUC5AC in the IL-13-treated OE and KD cells of circRNA_0021727 and miRNA_145_5p were detected by ELISA. The results showed that the protein contents of GM - CSF, EOTAXIN, and MUC5AC in the circRNA_0021727 OE group were significantly higher than those in the control group, while the contents in the circRNA_0021727 KD group were significantly lower than those in the control group (**Figures 3A–C**). The protein contents of GM - CSF, EOTAXIN, and MUC5AC in the miRNA_145_5p OE group were lower than those in the control group, and the contents in the miRNA_145_5p KD group were significantly higher than those in the control group (**Figures 3D–F**).

### circRNA_0021727 inhibits the expression of miRNA_145_5p and promotes the expression of ADAM12

**Figure 4:**
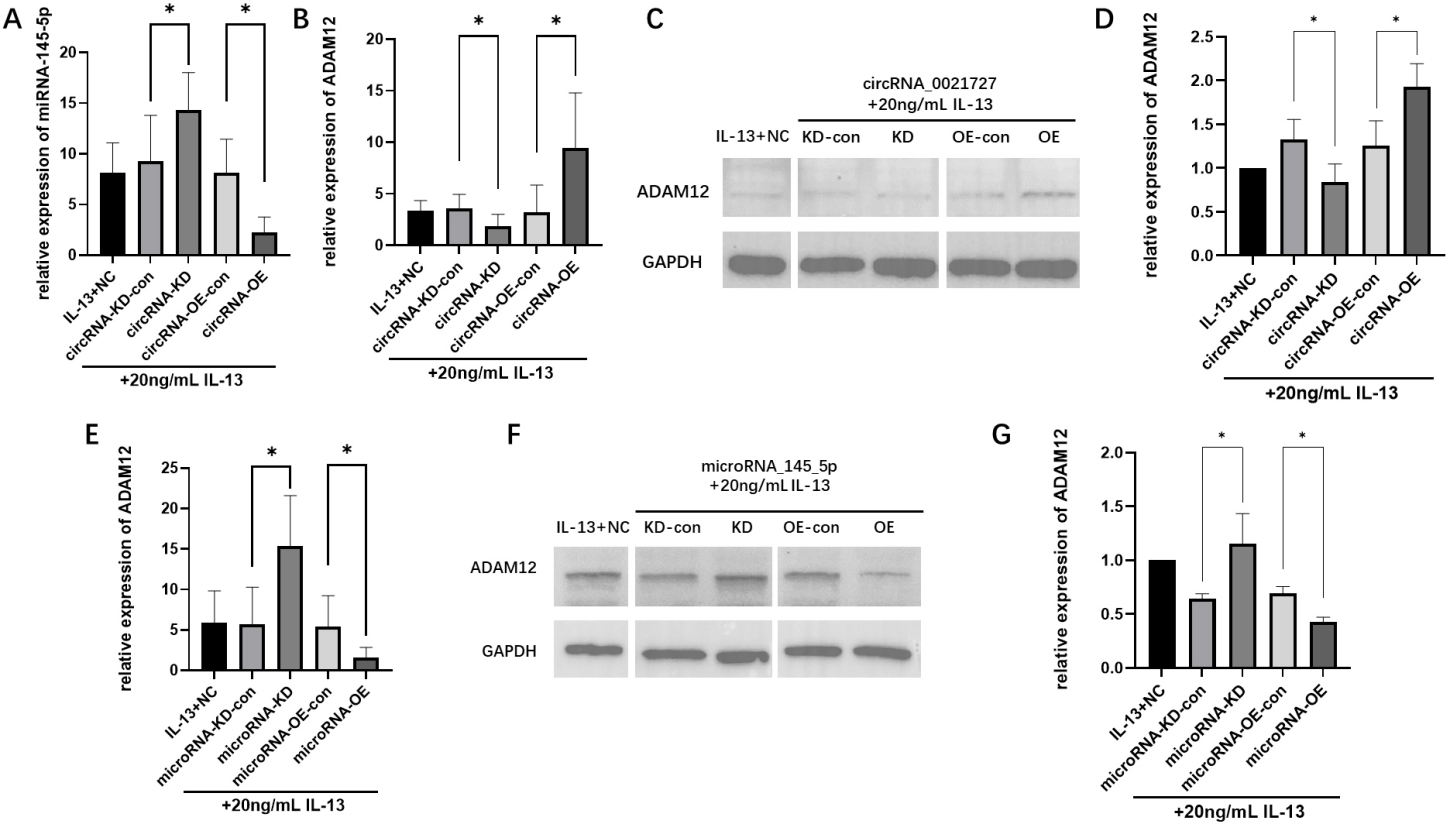
Expression levels of microRNA_145_5p and ADAM12 at mRNA and Protein Levels in circRNA_0021727 or microRNA_145_5p Overexpression and Knockdown Cells Treated with IL 13. In circRNA_0021727 OE cells, miRNA_145_5p expression was significantly lower than in controls, while in KD cells, it was significantly higher **(A)**. On the contrary, ADAM12 expression was significantly upregulated in OE cells and downregulated in KD cells compared to controls **(B)**. Western blot (WB) analysis confirmed that ADAM12 protein levels were elevated in OE cells and reduced in KD cells relative to controls **(C)**. The densitometric analysis also confirmed that the overexpression and knockdown cells showed significant differences compared with their respective control groups, and the trend was consistent with the WB results **(D)**. In miRNA_145_5p OE cells, ADAM12 expression was significantly lower than in controls, while in KD cells, it was significantly higher **(E)**. WB results were consistent with mRNA changes, showing decreased ADAM12 protein in OE cells and increased protein in KD cells compared to controls **(F)**. Consistent with the WB findings, the densitometric analysis revealed significant differences in the overexpression and knockdown cells compared with their corresponding control groups **(G)**. OE, overexpression; KD, knockdown; con, control; NC, negative control. *P < 0.05.

The IL-13-treated circRNA_0021727 OE and KD cells were collected, and RNA and proteins were extracted. After reverse-transcribing the extracted RNA, the expression levels of miRNA_145_5p and ADAM12 were detected by RT-qPCR. The results showed that in the circRNA_0021727 OE group, the expression level of miRNA_145_5p was significantly lower and the expression level of ADAM12 was significantly higher compared with the control group. In the KD group, the expression level of miRNA_145_5p was significantly higher and the expression level of ADAM12 was significantly lower compared with the control group, and the differences were statistically significant (**Figures 4A, B**).

The extracted proteins were detected by Western blot. It was found that the protein content of ADAM12 in the circRNA_0021727 OE group was higher than that in the control group, while in the KD group, it was lower than that in the control group (**Figure 4C**). The densitometric analysis also confirmed that the overexpression and knockdown cells showed significant differences compared with their respective control groups, and the trend was consistent with the WB results (**Figure 4D**).

### miRNA_145_5p inhibits the expression of ADAM12

The IL-13-treated miRNA_145_5p OE and KD cells were collected, and RNA and proteins were extracted from them. After reverse - transcribing the extracted RNA, the expression level of ADAM12 was detected by RT-qPCR. The results showed that the expression level of ADAM12 in the miRNA_145_5p OE group was significantly lower than that in the control group, while in the KD group, it was significantly higher than that in the control group. The differences were statistically significant (**Figure 4E**).

The extracted proteins were analyzed by Western blot. The results showed that the protein content of ADAM12 in the miRNA_145_5p OE group was lower than that in the control group, while in the KD group, it was higher than that in the control group (**Figure 4F**). Consistent with the WB findings, the densitometric analysis revealed significant differences in the overexpression and knockdown cells compared with their corresponding control groups (**Figure 4G**).

## Discussion

The treatment of ECRS, a refractory form of CRS, is extremely challenging. Even with successful surgical intervention and standardized postoperative medication, many patients still experience recurrence (3, 15). However, the etiology of ECRS is intricate, and the exact underlying mechanisms remain incompletely elucidated to date (16). Therefore, in-depth research into the mechanisms of ECRS development and progression, as well as the exploration of relevant pathogenic cytokines and chemokines, is of great significance for guiding the diagnosis and treatment of ECRS in the future.

In this study, we analyzed differential RNA expressions between the nasal polyp tissues of ECRS patients and the inferior turbinate tissues of non-CRS controls. Compared to controls, the expression levels of circRNA_0021727 and ADAM12 were significantly increased (P<0.05), while miRNA_145_5p expression was significantly decreased (P<0.05) in ECRS polyp tissues. These findings are consistent with our previous results in CRSwNP patients (14), suggesting the possible existence of a circRNA_0021727–miRNA_145_5p–ADAM12 regulatory pathway. At present, the known regulatory functions of circRNA_0021727 are primarily studied in oncology. It has been shown to promote the proliferation, migration, and invasion of tumor cells in esophageal squamous cell carcinoma, colon cancer, breast cancer, and glioblastoma (17–20). However, its role in non-neoplastic diseases remains unexplored. miRNA_145_5p has been reported to induce apoptosis of airway epithelial cells, reduce inflammation in COPD, and decrease susceptibility to airway inflammation (21, 22). ADAM12 is highly expressed in asthma patients (23), where it contributes to bronchial airway remodeling mucosal development, promoting chronic asthma symptoms (24, 25). In this study, overexpression of circRNA_0021727 significantly decreased miRNA_145_5p expression in epithelial cells while increasing both the mRNA and protein levels of ADAM12. Conversely, knockdown of circRNA_0021727 led to the opposite effects, indicating that circRNA_0021727 regulates miRNA_145_5p and ADAM12 upstream by inhibiting miRNA_145_5p expression and promoting ADAM12 expression. Similarly, overexpression of miRNA_145_5p reduced ADAM12 mRNA and protein levels, while its knockdown resulted in the opposite effects, demonstrating that miRNA_145_5p negatively regulates ADAM12. These findings support the existence of a circRNA_0021727–miRNA_145_5p–ADAM12 regulatory pathway in ECRS.”.

GM-CSF is a particularly important cytokine for regulating the development of eosinophils. It can promote the maturation of eosinophils, facilitate their release into the bloodstream, and enhance tissue infiltration (26). EOTAXIN has the function of activating and recruiting inflammatory cells, especially having a significant effect on eosinophils. It can stimulate the migration of eosinophils into tissues and enhance the inflammatory response by prolonging the survival time of eosinophils (5). The expression of MUC5AC can cause mucus obstruction and ciliary dysfunction, providing an environment for microbial colonization and exacerbating nasal mucosal inflammation (10). Therefore, the expression levels of GM-CSF, EOTAXIN, and MUC5AC in primary cells can reflect the severity of inflammation in ECRS. In the inflammatory environment created by IL-13, when circRNA_0021727 was overexpressed, the expression levels of GM-CSF, EOTAXIN, and MUC5AC in nasal epithelial cells were significantly increased, while they decreased when circRNA_0021727 was knocked down. It suggests that circRNA_0021727 promotes inflammation in ECRS. When miRNA_145_5p, which is downstream of circRNA_0021727, was overexpressed, the expression levels of GM-CSF, EOTAXIN, and MUC5AC in the cells decreased, and increased when miRNA_145_5p was knocked down. This therefore indicates that miRNA_145_5p can inhibit inflammation in ECRS.

These results confirm the existence of the circRNA_0021727-miRNA_145_5p-ADAM12 regulatory pathway in ECRS. In ECRS, circRNA_0021727 expression is elevated, promoting the production of inflammation-related factors, while, miRNA_145_5p inhibits the inflammatory response. Therefore, circRNA_0021727 and miRNA_145_5p may serve as potential diagnostic markers and therapeutic targets for ECRS.

The identification and functional validation of the circRNA_0021727-miRNA_145_5p-ADAM12 regulatory axis hold significant translational implications for ECRS management. Our previous work (14) has already indicated a positive correlation between circRNA_0021727 expression and key clinical indicators of ECRS, including eosinophil parameters, TNF-α, IFN-γ, and tissue eosinophil counts. These associations strongly suggest that molecular alterations within this axis are not merely cellular phenomena but are deeply intertwined with the clinical manifestation and severity of the disease. For instance, elevated circRNA_0021727 or suppressed miRNA_145_5p levels could potentially serve as valuable biomarkers for predicting disease activity, assessing therapeutic efficacy, or identifying patients at higher risk for recurrence. Future investigations are warranted to systematically correlate the expression profiles of circRNA_0021727, miRNA_145_5p, and ADAM12 with established clinical parameters such as objective disease severity scores, subjective symptom burden, and responses to various medical or surgical interventions. Such studies would solidify the clinical utility of this axis as a diagnostic tool, a prognostic indicator, and a potential target for novel precision therapies aimed at mitigating eosinophilic inflammation in ECRS.

This study has some limitations. One concerns the choice of control tissue. Since nasal polyp tissues often originate from the ethmoid sinus and middle meatus, using nasal mucosal tissue adjacent to the middle meatus as a control may be more suitable for investigating the pathogenesis of CRSwNP than the inferior turbinate (IT). As a result, some researchers prefer to use the MT or ET as controls. A study has shown that IT has a distinct glandular composition compared to the middle turbinate (MT) (27), and the ethmoid tissue (ET) may better reflect the inflammatory status of the CRS (28). However, recent findings suggest that the IT may exhibit a similar inflammatory profile to the nasal mucosa adjacent to the middle meatus (29). Moreover, Huang et al. report that the IT, MT, uncinate process (UP), and ET are all covered by pseudostratified ciliated columnar epithelium (30), and the expression patterns of tight junction molecules are similar across epithelial cells from different tissue origins. Consequently, many studies also use the IT as a control (31, 32). In addition, this study lacks *in vivo* experiments to further elucidate the mechanisms of circRNA_0021727 and validate the functions of ADAM12 in ECRS. These investigations will be conducted in future studies.

Our study confirms the overexpression of the circRNA_0021727-miRNA_145_5p-ADAM12 regulatory pathway in the nasal mucosa of patients with ECRS. circRNA_0021727 plays a crucial role in promoting inflammation in ECRS, contributing to its occurrence and progression.

## Data Availability

The raw data supporting the conclusions of this article will be made available by the authors without undue reservation.
